# Caregiver Burden in Parkinson Disease: A Scoping Review of the Literature from 2017-2022

**DOI:** 10.1177/08919887231195219

**Published:** 2023-08-08

**Authors:** Whitley W. Aamodt, Benzi M. Kluger, Miray Mirham, Anna Job, Samantha E. Lettenberger, Philip E. Mosley, Sandhya Seshadri

**Affiliations:** 1 Department of Neurology, 6572 University of Pennsylvania, Philadelphia, PA, USA; 2 Translational Center of Excellence for Neuroepidemiology and Neurology Outcomes Research, 6572 University of Pennsylvania, Philadelphia, PA, USA; 3 Department of Neurology, 6927 University of Rochester, Rochester, NY, USA; 4 School of Medicine, 6927 University of Rochester, Rochester, NY, USA; 5 6927 University of Rochester, Rochester, NY, USA; 6 Center for Health & Technology, 6927 University of Rochester, Rochester, NY, USA; 7 School of Medicine, University of Queensland, Herston, QLD, Australia

**Keywords:** caregiver, burden, strain, Parkinson disease

## Abstract

Caregiver burden is a term that refers to the adverse effect of caregiving on the physical, emotional, social, spiritual, and financial well-being of the caregiver. Caregiver burden is associated with providing care to an individual with a chronic illness or disability, and the unique symptoms of Parkinson disease (PD) can amplify a patient’s needs and reliance on others, leading to adverse outcomes for patients and their caregivers. In this scoping review of the literature from January 2017 through April 2022 that included 114 studies, we provide an updated, evidence-based summary of patient and caregiver-related factors that contribute to caregiver burden in PD. We also describe the impact of caregiver stress and burden on caregivers based on qualitative research studies and review recent interventions to mitigate burden. By providing clinical updates for practitioners, this review is designed to improve recognition of caregiver burden in the post-pandemic era and foster the development of targeted interventions to reduce caregiver burden in PD.

## Introduction

Parkinson disease (PD) is the second most common neurodegenerative disorder and affects over 6 million persons worldwide.^1^ PD has no known cure and leads to progressive physical and cognitive impairment. Although symptomatic treatments are available, symptom progression in later stages of disease can limit independence, requiring patients to increasingly rely on caregivers for assistance.^2^ Caregiver tasks can range from medication management to help with activities of daily living. PD has long been associated with high rates of caregiver burden, a phenomenon linked to adverse health outcomes for both patients and caregivers.^3^

Although no formal definition of caregiver burden exists, Zarit and colleagues proposed the following definition in their seminal 1986 study: “The extent to which caregivers perceive that caregiving has had an adverse effect on their emotional, social, financial, physical, and spiritual functioning.”^4^ Not surprisingly, the act of caregiving is an individualized experience with different thresholds for burden in persons from varied backgrounds. Caregiving can be perceived as an emotionally rewarding process that affirms family ties, honors loved ones, and preserves family resources^5^ as well as an overwhelming task. The balance between the positive and negative aspects of caregiving can shift over time. In the general population, female sex, low educational attainment, social isolation, financial stress, and more caregiving hours increase risk of burden, among other factors.^5^ A patient’s diagnosis and associated symptoms also correlate with caregiver burden, and the motor and non-motor symptoms unique to PD can amplify patient needs and reliance on caregivers. Symptomatic treatments for PD, including pharmacologic therapy and functional neurosurgery, can also lead to side effects, further exacerbating caregiver strain. Thus, identifying the contributors to caregiver burden in PD is a crucial first step in combatting this often underrecognized issue.

### Significance of This Review

Numerous studies have explored patient and caregiver factors that contribute to caregiver burden. In 2017, Mosley and colleagues published a comprehensive review of caregiver burden in PD.^6^ While this review appraised the predictors and correlates of caregiver burden for practicing clinicians and provided a description of instruments used to measure caregiver burden in PD, over 150 papers have been published on this topic in the last 5 years, which parallels the rise in unpaid caregivers from 43.5 million in 2015 to 53 million in 2020 in the United States (US) alone.^7^ In 2021, Hulshoff and colleagues also published a scoping review of longitudinal studies that explored caregiver burden, needs, and coping in PD, but only PubMed was queried and 22 studies were reviewed though May 2020.^8^ In recent years, forced isolation during the COVID-19 pandemic has exacerbated rates of caregiver burden in PD, prompting new approaches for management and intervention.^9,10^ This scoping review contributes to the existing literature by providing clinical updates for PD practitioners and researchers. These updates will not only allow for improved recognition of caregiver burden in the post-pandemic era, but also encourage the development of new interventions to improve PD caregiver outcomes.

### Aims

We aim to provide an updated, evidence-based summary of factors that contribute to caregiver burden in PD based on literature from 2017-2022. Results from this review are grouped by category and divided into 4 sections: 1) patient-related contributors, 2) caregiver-related contributors, 3) impact of caregiver stress and burden on caregivers, and 4) existing therapies and interventions to reduce caregiver burden in PD.

## Methods

We conducted a scoping review of caregiver burden in PD to describe factors related to burden and identify knowledge gaps in the field. Unlike systematic reviews that appraise and synthesize data related to a focused research question, scoping reviews are an ideal tool to survey a body of literature and provide an overview of available evidence that may inform future systematic reviews.^11^ Our preliminary research questions were three-fold:

1. What patient and caregiver factors contribute to caregiver burden in PD?
2. What is the impact of caregiver stress and burden on PD caregivers?
3. What existing interventions have addressed caregiver burden in PD?

Our protocol was developed in accordance with the Preferred Reporting Items for Scoping Reviews (PRISMA-ScR) guidelines^12^ and summarized below. There were no protocol deviations.

### Information Sources and Eligibility Criteria

Based on guidance from prior literature, we queried PubMed and PsychINFO to provide a balance of breadth and specificity.^6^ We also included EMBASE to capture articles published in international journals and CINAHL to capture publications from nursing and allied health journals that have not been included in prior reviews. The following search terms were used to find peer-reviewed research articles published between January 1, 2017 and April 1, 2022:

• PubMed: ((parkinson disease[mh]) AND caregiver[mh]) AND (burden OR anxiety OR depression OR distress OR stress OR strain) AND (Humans[Mesh])
• PsychINFO: 1) MeSH: depression, 2) MeSH: anxiety, 3) Any Field: burden, 4) Any Field: stress, 5) Any Field: strain, 6) 1 OR 2 OR 3 OR 4 OR 5, 7) MeSH: caregivers, 8) MeSH: parkinson disease, 9) 6 AND 7 AND 8 AND 9
• EMBASE: (‘parkinson disease'/exp OR ‘parkinson disease’ OR (parkinson AND (‘disease'/exp OR disease))) AND (‘caregiver'/exp OR caregiver) AND (‘burden'/exp OR burden OR ‘anxiety'/exp OR anxiety OR ‘depression'/exp OR depression OR ‘distress'/exp OR distress OR ‘stress'/exp OR stress OR ‘strain'/exp OR strain)
• CINAHL: Parkinson disease AND caregiver AND burden OR anxiety OR depression OR distress OR stress OR strain

At the time of the initial search, we excluded non-peer reviewed manuscripts, letters to the editor, editorials, study protocols, conference abstracts, and articles that were not authored in English. Following each search, duplicates were removed, and references of review articles were hand searched for additional papers. Next, all remaining abstracts were reviewed by authors (WWA, SS), and papers were categorized into relevant sections outlined below in a database that summarized key elements from each study, including citation, country of origin, objective/aims, study population, design, results, and conclusions. Database development was an iterative process, with refinement of categories as needed. Following categorization, full texts were reviewed by authors (WWA, SS, MM, AJ, SL) and included in the study if papers were deemed relevant to caregiver burden in PD. Articles were excluded if they were beyond scope or did not relate to the research questions (e.g., no analysis of caregiver burden, no outcome measure of caregiver burden, not relevant to PD, involved development or validation of a caregiver burden inventory or outcome metric), or if the full-text was unavailable. Articles of unclear significance were discussed in consensus meetings to determine relevance, at which time they were marked for inclusion or exclusion. Because our primary objectives were to describe contributors to caregiver burden in PD and identify knowledge gaps in the field, we did not perform a critical appraisal of included studies, which was considered beyond the scope of this broad narrative synthesis. Following the approach used by Mosley and colleagues,^6^ we present our data in a thematic manner by section.

## Results

### Summary of Search Results

**Figure 1.**
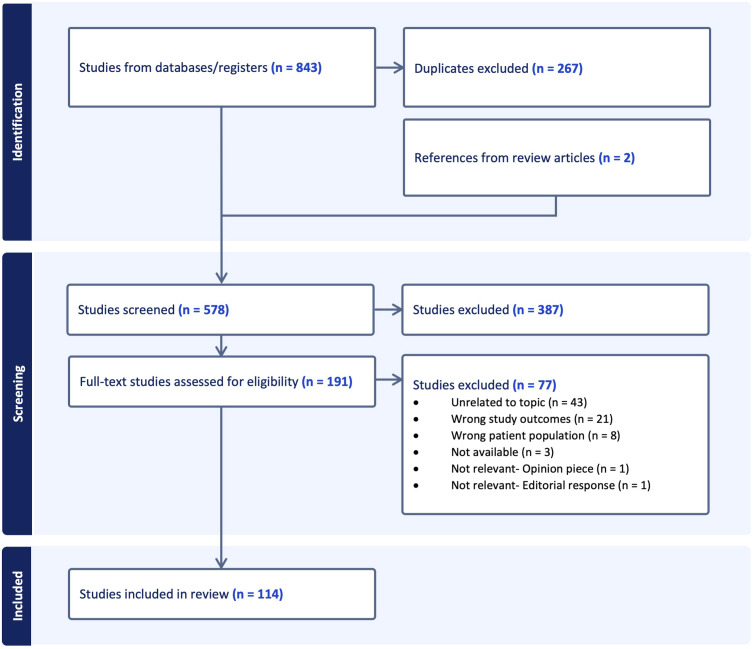
Papers selected for final inclusion. PRISMA flowchart created using Covidence.

After querying PubMed, PsychINFO, EMBASE, and CINAHL using the search terms noted above, 843 references were exported for screening, of which 267 duplicates were removed. Five review articles returned during this initial search, and 2 additional papers were discovered after handsearching their reference lists. We then screened 578 papers against title and abstract, of which 387 were excluded based on relevance to the research questions. Lastly, 191 full-text papers were assessed for eligibility, and 114 were included in the final review. This process and relevant exclusion criteria are summarized in Figure 1.

### Presentation of Results

Results below are organized based on the following categories: (1) predictors and contributors to burden, including patient, caregiver, and societal and cultural factors, (2) impact of caregiver stress and burden on caregivers, and (3) existing therapies and interventions to mitigate caregiver burden in PD. When taken together, these data provide an updated summary that can be used by both clinicians and researchers to improve recognition of caregiver burden and develop new interventions to improve quality of life for PD caregivers.

## Predictors and Contributors to Burden

### Patient Factors

#### Motor Symptoms

PD is characterized by progressive motor symptoms, including bradykinesia, rigidity, tremor, and gait instability that can impair functioning and lead to falls. Although these symptoms may respond to dopaminergic therapy in the initial stages of disease, symptoms can worsen in between doses of medication, a phenomenon known as “off” time, or become refractory to treatment. Patients with moderate to advanced PD can also experience unpredictable motor fluctuations and abnormal movements, or dyskinesias, that develop following long-term use of dopaminergic therapies. Not surprisingly, prior studies have shown a positive association between PD duration, motor symptom severity, disability, Hoehn and Yahr stage, and caregiver burden,^6^ meaning that greater PD symptoms result in greater caregiver burden.^13^ Caregiver burden is also associated with higher mean levodopa equivalent daily dose and levodopa-related motor complications.^14^

Recent studies have largely confirmed these findings, and motor severity measured by the Unified Parkinson’s Disease Rating Scale (UPDRS) Part III significantly correlates with caregiver burden assessed by the Zarit Burden Inventory (ZBI)^15-17^ and PD Caregiver Burden Inventory.^18^ Higher scores on the UPDRS Parts II and IV, assessments of the motor aspects of daily living and motor fluctuations, respectively, also correlate with higher ZBI score.^14,19^ Of PD motor symptoms, akinesia and rigidity are more likely than tremor to contribute to caregiver burden.^17,20^ In a recent paper that explored the economic burden of PD “off” time in patients with motor fluctuations, caregivers of persons with PD “off” periods also reported lower productivity at work, more missed work days, and more lost income than caregivers of PD patients without “off” periods.^21^ These data suggest that PD motor symptoms and fluctuations contribute to financial strain in persons with PD and their caregivers, further increasing societal-level economic burden.^21^

#### Non-Motor Symptoms

Although PD diagnosis and staging are dependent on the presence of motor symptoms, non-motor symptoms like neuropsychiatric and sleep-related disturbances can significantly impact both patient and caregiver quality of life. Non-motor symptoms can also magnify physical disability, increase need for supervision, and affect a patient’s relationship with their caregiver.^6^ In prior studies, non-motor symptoms more strongly correlated with caregiver burden than motor symptoms when examined concurrently.^6,17^

#### Neuropsychiatric Symptoms

Depression, apathy, cognitive impairment, hallucinations, delusions, psychosis, agitation, and aggression commonly contribute to caregiver burden in PD.^22-26^ Depression and apathy, along with decreased empathy and emotion recognition deficits,^27-29^ have been associated with reduced caregiver well-being. In a recent study from Germany, caregiver burden was associated with alexithymia among persons with PD, a cognitive affective disturbance resulting in difficulty identifying and expressing emotions.^30^ The severity of cognitive impairment is also a consistent predictor of caregiver burden in PD,^31-34^ and disorientation and short-term memory deficits,^32^ attentional deficits,^34^ and low verbal fluency^35^ can negatively impact caregiver quality of life. Importantly, cognitive impairment can exacerbate neuropsychiatric symptoms in PD.^36^ While impulsive and compulsive behaviors contribute to caregiver distress at all PD stages,^37^ the impact of psychosis on caregiver burden in PD is greater when the care recipient has dementia and less insight into their behaviors.^38^ Agitation and disinhibition among PD patients, including physical and sexual aggression,^39^ can also intensify the emotional and psychological burden of caregiving.^38^

#### Sleep Disturbances

Patient sleep quality is a strong predictor of caregiver health-related quality of life, and poor sleep quality among persons with PD can lead to worse caregiver well-being.^40^ Nocturnal care demands also place additional strain on PD caregivers. In a recent study of 253 caregivers from the US and Mexico, PD sleep disturbances contributed to caregiver sleep problems, increased caregiver burden, and reduced life satisfaction.^41^ In a smaller study of 29 caregivers from Australia, poor sleep quality was also associated with increased caregiver burden, depression, anxiety, fatigue, poor quality of life, and poor sleep hygiene.^42^ Over time, these sleep disturbances may serve as a risk factor for caregiver depression.^6^

#### Age of Onset, Symptom Burden, Overall Disease Severity, and Gender

When comparing patients with different disease types, caregivers of persons with young-onset PD (YOPD) experience less strain than caregivers of persons with late-onset PD, which is largely attributed to slower rates of disease progression among YOPD patients.^43^

PD symptom burden has also been linked to caregiver burden. In a survey of caregivers from the US and Mexico, cluster analysis using domains from the UPDRS found that caregiver burden was greatest in caregivers of patients with severe diffuse symptoms and moderate symptoms accompanied by vocal changes and/or dysphagia.^44^ Dysphagia, in particular, has been shown to negatively impact caregiver well-being.^45^

Persons with advanced PD are highly dependent on their caregivers for support, and increasing physical dependence and reduced ability to complete activities of daily living can result in higher rates of caregiver burden.^46,47^ Interestingly, 1 study from Turkey did not find significant differences in caregiver burden between caregivers of patients with early and late-stage PD.^27^ However, these results were attributed to Turkey’s patriarchal society, in which females who perform the bulk of caregiving duties may perceive tasks as their familial obligation regardless of disease severity or stage.^27^

With regard to patient gender, caregivers who provide care to male patients have worse quality of life.^48^ The exact explanation for these gender differences is unknown, but more disabling symptoms among men, including dementia with psychosis and falls,^49^ may increase the emotional burden on caregivers and contribute to safety concerns. In another study using data from the Parkinson Foundation Parkinson’s Outcomes Project from 2009-2014, caregivers of male patients were more likely to report caregiver strain after controlling for age, disease stage, comorbidities, cognitive and mobility measures, and health-related quality of life.^50^

### Caregiver Factors

Numerous caregiver factors have been associated with caregiver burden in PD, including caregiver gender, relationship to the patient, and medical and psychiatric comorbidities.^6^ Caregiving hours are also associated with caregiver burden in PD,^6^ and the number of hours per day spent assisting and supervising PD patients increases the risk of caregiver burden.^24^

#### Gender Differences

In persons with PD, men are more likely than women to have a caregiver, and women more often serve as caregivers.^50^ In 1 study from Germany, there were no gender differences in task-specific caregiver burden.^51^ However, when comparing caregiver outcomes, female caregivers usually report worse quality of life.^24^ In prior studies, female care providers were twice as likely as male care providers to report exhaustion and damage to their health resulting from care provision in PD.^52,53^ Social constraints and time limitations were also more common among female caregivers,^52,53^ and female caregivers are more likely to suffer from anxiety or depression.^54^ In addition, spouses are more likely to report greater caregiver burden than other carers,^24,55^ and caregivers of female patients who are not spouses or partners report improved quality of life.^56^

#### Medical and Psychiatric Comorbidities

The act of caregiving can place a physical and psychological strain on caregivers, leading to increased medical and psychiatric comorbidities. In 1 study of 107 PD caregivers in the United Kingdom, 30 (26.1%) reported musculoskeletal conditions, 7 (6.1%) reported mental health problems, 5 (4.3%) reported respiratory conditions, 4 (3.5%) reported cancer, 4 (3.5%) reported diabetes mellitus, and 4 (3.5%) reported cardiovascular disease that impact their ability to perform caregiving duties.^57^ Few studies have explored pre-morbid psychiatric comorbidities among PD caregivers. However, as a consequence of caregiving, PD caregivers are also more likely to experience depression and anxiety than controls with high rates of other psychological symptoms that can further exacerbate burden.^58-62^ In PD caregivers from Iran, female sex, single status, and medical illnesses were associated with more psychiatric symptoms.^63^ In PD caregivers from Taiwan, depression was associated with fatigue and suicidal ideation.^64^ In the course of performing caregiving duties, PD caregivers also report more anticipatory grief, perceived criticism, and reduced satisfaction in their personal relationships.^65-68^ Resilience, however, may improve mental health symptoms, in part, among PD caregivers and has been associated with improved relationship satisfaction and well-being.^69-71^

### Societal and Cultural Factors

Societal and cultural factors, including the PD family unit and access to caregiving resources, can influence caregiver burden in PD. In Mexico, perceived family cohesion was associated with reduced caregiver burden,^72^ presumably because responsibility could be divided among multiple family members who may reside together. In another study comparing caregiver needs in the US and Mexico, PD caregivers in the US were at increased risk of poor emotional and community support, which contributed directly to caregiver anxiety and depression.^73^ In Mexico, caregivers had stronger emotional and community support, but the need for instrumental support, defined as tangible aid and service, generated more anxiety and depression.^73^

The impact of PD symptoms on caregiver burden in PD was particularly evident during the COVID-19 pandemic and exacerbated by social isolation, disruptions to healthcare delivery and other support services, increased symptomatic and psychosocial needs, and telemedicine limitations compared to in-person medical care.^10^ Pre-existing levels of anxiety, motor complications, and poor quality of life among PD patients, combined with the total number of lockdown hours per day, also had a major psychological impact on patients and caregivers by proxy.^74^ There was a higher prevalence of anxiety among patients with advanced PD and their caregivers during the pandemic,^75^ with many caregivers reporting increased levels of stress.^76^

Information access also plays a role in caregiver burden. For example, caregiver stress is associated with lack of knowledge and access to PD information^77,78^ and uncertainty about the future.^77-80^ Lack of medication information,^81^ knowledge on the cost of medications,^82^ and increased financial worries^79,80,82,83^ also make caregiving more challenging. In interpersonal interactions, caregiving stress is heightened by lack of understanding from family and social circles^77,84^ as well as perceived lack of information and support from healthcare teams.^77-79,85^

### Impact of Caregiver Stress and Burden on Caregivers

Relentless caregiving tasks can negatively impact caregivers’ physical and mental health. The need to provide continual support for activities of daily living, for example, limits a caregiver’s time for other preferred activities,^85-87^ decreases self-care,^83^ and increases social isolation. Recent studies utilizing qualitative research methods (e.g., semi-structured interviews, focus groups, and participant observation) highlight the impact of caregiving stress and burden on PD caregiver well-being. These results are organized based on the following categories: (a) impact on emotional health, (b) decreased self-care, (c) losses and disruptions in relationships, and (d) stigma and social isolation.

#### Impact on Emotional Health

Emotionally, caregivers experience depression, grief, loss, anxiety, distress, guilt,^77,81,86,88,89^ feel helpless and frustrated,^83,87^ and report sadness watching their loved one deteriorate.^77^ Caregiver stress is exacerbated by the experience of conflicting needs and emotions. Caregivers fear increased falls if not vigilant^81^ but feel guilty when relying on others to provide needed respite care. Caregivers also experience mixed emotions when considering moving a patient to a nursing home or other facility.^56^

#### Decreased Self-Care

The inability to leave the patient alone at home leads to increased physical and emotional fatigue.^56^ The lack of time for self-care and pursuit of their own interests,^83,86,87,90^ along with lack of sleep, makes the task of constant caregiving harder.^79,89,91^ Some caregivers perceive their own needs as less important as they embrace the role of being the person in charge and protector.^88^

#### Impact of Losses and Disruptions in Relationships

The shift in roles (from spouse to caregiver) also impacts the spousal relationship^77,86,91^ and in turn results in feelings of loss and sadness.^88^ Losses relate to the relationship itself, the identity of the caregiver, and the loss of future plans and hopes.^86-88,92^ The stress of altered relationships and roles is acutely experienced by spouses of patients with impulse control disorders, as they may feel shame in sharing these behaviors with others.^92^ Patients’ cognitive impairments also negatively impact relationships and result in heightened frustration, sadness, and loss of intimacy.^86,88,89^

#### Stigma and Social Isolation

Negative public attitudes concerning bowel and bladder issues in PD remain a source of stigma for caregivers and families.^93^ For example, physically caring for patients with lower urinary tract symptoms is challenging for female spouses who struggle to find coping strategies.^10,87,88,91^ Saliva, drooling, and dyskinesias experienced by patients can reduce their emotional well-being and lead to increased caregiver burden.^94^ Concerns about stigma among caregivers of patients with visual hallucinations also lead to delays in seeking help.^84^ Though caregivers desire social interactions, physical exhaustion, public perceptions of PD symptoms, and stigma lead to increased social isolation.^52,75,79^ Social isolation and caregiver burden also worsened during the pandemic due to social distancing.^9^

### Interventions

Between 2017-2022, 10 studies explored the impact of advanced PD therapies on caregiver burden, while 12 studies explored non-medical interventions to mitigate caregiver burden in PD. These non-medical interventions included 6 randomized controlled trials (RCTs), 3 pilot studies, and 3 quasi-experimental studies (Table 1). Medical and non-medical interventions can be divided into 6 broad categories summarized below: advanced patient therapies, cognitive or group therapies, psychoeducational interventions, self-management programs, community support groups and classes, and palliative care consultation.

**Table 1.** Summary of non-medical interventions addressing caregiver burden in Parkinson disease, 2017-2022.

Study	Intervention	Country	Sample Size	Design	Caregiver Outcome Measures	Results
Cognitive Therapies						
Wuthrich et al. (2019)^105^	CBT. Participants randomized to 10-week telephone based CBT program vs control group	Australia	n = 11 pwPD, n = 3 caregivers	RCT; data collected at baseline, 10 weeks, 1 month post-intervention	DASS, ZBI short form	Caregivers reported reduction in depressive symptoms and burden, though effect sizes were large
Mosley et al. (2021)^106^	CBT. Caregivers of pwPD s/p STN DBS received 8 60-minute sessions	Australia	n = 10 caregivers	Non-randomized pilot intervention; data collected at 0 and 3 months post-program	ZBI, PDQ-carer, GDS, GAI, RQI	Caregivers reported improved quality of life, reduced anxiety and burden at program completion; anxiety and burden benefits maintained at 3 months
Leroi et al. (2019)^107^	CST. pwPD-caregiver dyads randomized to CST vs usual treatment	UK	n = 76 patient-caregiver dyads (n = 15 PD-MCI, n = 40 PDD, n = 21 DLB)	Single-blind pilot RCT	DRS, SF-12, FCR, ZBI, RSS, EQ5D, HADS, RSS, BRS	Caregivers in CST vs usual care groups reported improved quality of life (EQ5D, p = .048), relationship quality (RSS, p = .020), and more anxiety (HADS, p-.112); burden and stress also improved, but results not significant
Group Therapy						
Poyner-Del Vento et al. (2018)^108^	Participants randomized to 8 sessions of group therapy	USA	n = 7 female caregivers of military veterans with PD	Pre/post pilot intervention; data collected during sessions 1, 4, and 8, 1-month follow up completed within 4-8 weeks post-treatment	DASS, ZBI, QMI	No statistically significant change was observed in caregiver burden or perspective-taking in all 7 caregivers. 2 demonstrated declines in psychological distress, 2 demonstrated increases in relationship satisfaction, and 1 saw changes in both domains
Psychoeducational Interventions						
Navarta‐Sánchez et al. (2020)^109^	PwPD and caregivers received a 9-week psychoeducational intervention, controls received a 5-week educational program	Spain	Intervention group: n = 51 pwPD, n = 37 caregivers Control group: n = 59 pwPD, n = 53 caregivers	Quasi-experimental study; data collected pre/post intervention and at 6 month follow-up	SQLC	No caregivers reported improved quality of life, though caregivers in both groups reported improved psychosocial adjustment to illness and coping skills
Bagheri et al. (2019)^110^	Family-centered empowerment intervention. Caregivers were randomized to 4 45-minute training sessions: 2 sessions reviewed PD complications and reduction strategies, 2 sessions asked caregivers to teach course materials to other family members	Iran	n = 30 pwPD, n = 30 caregivers	RCT; data collected at randomization and 1.5 months post-intervention	ZBI	Caregivers in the intervention vs control group reported improved burden (ZBI scores dropped 25.1±13.9 points vs .6±3.1 points, respectively). The intervention group also showed a significant difference between total care burden score before and after the intervention (P < .001)
Self-Management Programs						
Lyons et al. (2021)^111^	pwPD-caregiver dyads participated in a community-based self-management workshop	USA	n = 19 (pwPD-caregiver dads), n = 20 control dyads	Quasi-experimental two-wave design; data collected after 7 weeks of follow-up	CES-D, MCSI	Caregivers in the intervention group reported fewer depressive symptoms and more engagement in strength-based activities and self-efficacy to support their partner; there was minimal improvement in care strain
Hellqvist et al. (2020)^112^	pwPD and caregivers attended the Swedish National Parkinson School (7 2-hour small group sessions with educational material, discussion, and 15 minute relaxation exercise) vs standard care	Sweden	Intervention group: n = 48 pwPD, n = 30 caregivers Control group: n = 44 pwPD n = 25 caregivers	Quasi-experimental case-control study; data collected pre- and post-intervention	ZBI	There were no significant differences between intervention and control groups. Baseline ZBI scores in intervention group [7 (3-13)], follow-up [8 (3.25-12.75)]. Baseline ZBI scores in control group [6 (.75-12.5)], follow-up [5 (2-13.25)].
Shah-Zamora et al. (2021)^113^	pwPD-caregiver dyads enrolled in 9-week mindfulness-based stress reduction course	USA	n = 26 pwPD, n = 11 caregivers	Pre/post pilot intervention; data collected at screening, end of course, 3 month follow-up	MASS, ZBI	Caregivers experienced an increase in mindfulness (p = .02), but no change in overall caregiver burden (*P* = .239)
Community Support Groups and Classes						
Tamplin et al. (2020)^116^	PwPD randomized to ParkinSong group or non-singing control conditions (painting, dancing, tai chi, peer support group) for 12 months; sessions lasted 2 hours and were conducted weekly and monthly	Australia	n = 75 pwPD, n = 44 caregivers	RCT with 4 arms, data collected at baseline, months 3 and 12	QPCR, DASS, EQ5D	Caregivers who attended weekly ParkinSong groups reported improvements in depression, anxiety and stress. There were no significant differences between groups for caregiver quality of life or perceived relationship quality
*Palliative Care Consultation*						
Kluger et al. (2020)^117^	pwPD and caregivers randomized to outpatient palliative care (neurologist, social worker, chaplain, nurse) vs stndard care (neurologist and PCP)	USA	n = 210 pwPDRD, n = 75 caregivers	RCT; data collected 6 months post-intervention	ZBI	There was a significant reduction in caregiver anxiety, improvement in spiritual well-being, and reduction in caregiver burden at 12 months
Veronese et al. (2017)^118^	Participants randomized to immediate PC visit or PC visit following 16-week waiting period	Italy	n = 50 patients (n = 16 PD, n = 16 ALS/MND, n = 18 MS), n = 45 caregivers	RCT, phase II pilot; data collected at baseline and 16 weeks	CBI	Patients reported improvement in physical symptoms and quality of life, caregiver burden was not impacted

Abbreviations (alphabetical): ALS, amyotrophic lateral sclerosis; BRS, Brief Resilience Scale; CBI, Caregivers Burden Inventory; CBT, cognitive behavioral therapy; CES-D, Center for Epidemiologic Studies-Depression; CST, cognitive stimulation therapy; DASS, Depression, Anxiety and Stress Scale; DBS, deep brain stimulation surgery; DLB, dementia with Lewy bodies; DRS, Dyadic Relationship Scale; EQ5D, EuroQoL-5D; FCR, Family Caregiving Role Scale; GAI, Geriatric Anxiety Inventory; GDS, Geriatric Depression Scale; HADS, Hospital Anxiety and Depression Scale; MASS, Mindful Attention Awareness Scale; MCI, mild cognitive impairment; MCSI, Multidimensional Caregiver Strain Index; MND, motor neuron disease; MS, multiple sclerosis; PDD, Parkinson disease dementia; PDQ, Parkinson’s Disease Questionnaire; pwPD, persons with Parkinson disease; pwPDRD, persons with Parkinson disease and related disorders; QMI, Quality of Marriage Index; QPCR, Quality of Carer Patient Relationship; RSS, Relationship Satisfaction Scale; RCT, randomized controlled trial; RQI, Relationship Quality Index; SF-12, Short Form-12 Health Survey; SQLC, Scale of Quality of Life of Caregivers; STN, subthalamic nucleus; UK, United Kingdom; USA, United States of America; ZBI, Zarit Burden Interview.

### Advanced Patient Therapies

Persons with PD are considered candidates for advanced therapies when motor symptoms are inadequately controlled with oral dopaminergic therapy due to disease progression or medication side effects. Enteral carbidopa/levodopa and deep brain stimulation (DBS) surgery are common surgical options, and recent studies have explored the impact of these therapies on caregiver burden given their ability to improve PD symptoms.

#### Enteral Carbidopa/Levodopa

Duodopa® and Duopa^TM^ are enteral suspensions of carbidopa/levodopa gel that are pumped continuously into the jejunum. These suspensions can improve both motor and certain non-motor symptoms in persons with PD, and recent studies have found a significant reduction in caregiver burden at 6 months^95^ and 12 months^96^ post-procedure. In studies from Italy, caregivers of PD patients who received carbidopa/levodopa intestinal gel also had greater reductions in caregiver burden than caregivers of PD patients who received subcutaneous apomorphine infusions or standard care,^97^ and had more time to perform family or household duties and engage in leisure activities.^98^ A recent study also showed mild improvement in caregiver anxiety 6 months post-procedure, but improvement in patient quality of life did not correlate with overall improvement in caregiver quality of life.^99^ This finding could be explained by the small sample size (n = 16) or short follow-up period; however, authors noted that quality of life improvements for patients with advanced PD may not immediately translate into improved quality of life for caregivers.^99^ Further longitudinal studies are needed.

#### Deep Brain Stimulation Surgery

DBS involves the implantation of leads into 1 of 2 locations in the basal ganglia based on patient symptomatology: subthalamic nucleus (STN) or globus pallidus interna. Leads are then connected to a battery in the chest wall, which provides electrical stimulation to ameliorate the motor symptoms of PD. Although DBS can significantly improve patient quality of life, prior studies suggest that caregiver burden may remain unchanged or worsen after DBS surgery, which could relate to the persistence of non-motor symptoms, stimulation-induced psychiatric symptoms, or sudden changes in the caregiver role.^6^ In 1 study from Spain, caregiver burden was compared between caregivers of DBS patients and PD patients receiving usual medical therapy.^100^ Consistent with prior research, treatment with DBS was not associated with lower caregiver burden.^100^ In another study from the Netherlands, caregiver burden was similar between pre- and post-operative assessments after 1 year.^101^ Two recent studies also explored caregiver burden after STN DBS, and caregiver burden worsened after 2 years of follow-up,^102^ which may be partly explained by post-operative neuropsychiatric symptoms.^103^ These findings emphasize the importance of assessing caregiver burden before functional neurosurgery with continued monitoring in the post-operative period. Lastly, a recent systematic review summarized patient and caregiver factors associated with improved caregiver well-being after DBS surgery.^104^ A favorable patient profile included younger age at disease onset, shorter disease duration, lower medication requirements, and fewer neuropsychiatric symptoms, while protective caregiver factors included higher pre-operative quality of life, younger age, fewer neuropsychiatric symptoms, and a more favorable pre-operative caregiver-patient relationship.^104^

### Non-Medical Interventions

#### Cognitive or Group Therapies

Cognitive therapies, such as cognitive behavioral therapy (CBT) and cognitive stimulation therapy (CST), may improve caregiver burden in PD, but data from group interventions are mixed. In 1 study from Australia, CBT delivered by phone improved depression, anxiety, and caregiver burden in 3 PD caregivers.^105^ Similarly, in another pilot study from Australia, ten STN DBS caregivers received 8 60-minute sessions of CBT in the post-operative period.^106^ Caregiver burden and anxiety were significantly reduced after completion of the program and benefits were maintained for at least 3 months, suggesting that short-course CBT may be particularly beneficial for DBS caregivers.^106^

In a RCT from the United Kingdom, PD caregivers were trained to deliver CST to PD patients with cognitive impairment or dementia.^107^ CST involves participating in cognitively stimulating and engaging activities at home. After 12 weeks, caregivers reported reduced burden, stress, and improved relationship quality.^107^ These results suggest that combined patient-caregiver interventions may be helpful for caregiver well-being.

Lastly, 1 study explored whether individual participation in the Caregivers' Attachment and Relationship Education (CARE) group therapy setting was effective in addressing the mental health needs of 7 PD caregivers.^108^ Although most caregivers enjoyed participating in the CARE program and expressed more willingness to seek out community resources, no statistically significant change was observed in caregiver burden.^108^

#### Psychoeducational Interventions

Psychoeducational interventions have also been shown to improve psychosocial adjustment and coping skills among caregivers.^109^ In a RCT from Iran, 30 PD caregivers were randomized to a “family-centered empowerment” intervention that involved 4 45-minute training sessions: 2 sessions that reviewed the perceived threat of PD complications and strategies to reduce them and 2 sessions in which primary caregivers were asked to teach course materials to other family members and review course content.^110^ Caregivers who received the family-centered empowerment training reported less caregiver burden 1.5 months after program completion.

#### Self-Management Programs

Self-management interventions have also shown improvements in PD caregiver quality of life, though data is overall mixed. In 1 pilot study, patient-caregiver dyads completed the “Strive to Thrive” self-management program.^111^ Spouses who completed the program reported fewer depressive symptoms, greater improvement in their engagement with mental relaxation techniques and strength-based activities, and greater self-efficacy to support their care recipient.^111^ In contrast, caregiver burden was measured following participation in the Swedish National Parkinson School, a self-management program designed for patients and family members that involved 7 2-hour sessions and relaxation exercises.^112^ Caregivers who completed the program reported no change to slight improvements in overall burden.^112^ Lastly, another study assessed the impact of a 9-week mindfulness-based stress reduction course in persons with PD and their caregivers.^113^ While this course produced a sustained improvement in mindful awareness among caregivers, there was no reduction in overall caregiver burden.^113^ When taken together, these data suggest that structured programming may reduce caregiver burden in PD,^114^ but self-directed stress reduction courses show more limited benefit than psychoeducational interventions.

#### Community Support Groups and Classes

When exploring the intervention needs of PD family members in Germany, the majority of caregivers believed they would benefit from group intervention.^115^ Group interventions can also teach patients and caregivers a new skill. One small study explored the effect of ParkinSong group singing classes on PD communication and patient and caregiver well-being. Caregivers who attended ParkinSong classes with their loved ones showed greater reduction in stress and depression.^116^

#### Palliative Care Consultation

Palliative care interventions may also mitigate caregiver burden in PD, though data are not robust. In 1 study comparing specialist palliative care and standard care, there was a significant reduction in caregiver anxiety, improvement in spiritual well-being, and reduction in caregiver burden at 12 months.^117^ However, in a second study, there was no difference in overall caregiver burden between the intervention and control groups.^118^ One limitation may involve the use of outcome measures that lack clinometric responsiveness to detect changes related to the disease or intervention.^119^

## Conclusions

As PD progresses over time, many patients with declining motor and cognitive function require assistance from caregivers to perform instrumental and basic activities of daily living. Studies have shown that PD patients with caregivers report improved quality of life,^120^ highlighting the importance of caregiver engagement on patient outcomes. Despite benefits to patients, caregivers can experience significant challenges in the course of their caregiving duties, including worsening anxiety and depression, declining health, and poor overall quality of life. Because PD is an age-related disorder with a mean age at diagnosis of 70.5 years,^121^ these challenges can be particularly detrimental to aging caregivers who often need support themselves. Caregiver burden also has larger societal consequences, and burden is considered a risk factor for healthcare utilization^122^ and nursing home placement/institutionalization^123^ among persons with PD. In this scoping review, we describe the patient-related and caregiver-related factors that contribute to caregiver burden in PD to improve recognition of this often overlooked phenomena. In addition, we summarize caregiver perceptions of stress and burden in PD and highlight recent caregiver interventions that may serve as models for future studies.

Similar to data from prior reviews, caregiver burden and reduced caregiver quality of life in PD are closely associated with the severity of both motor and non-motor symptoms, including akinesia, rigidity, dysphagia, cognitive impairment, mood disorders, and sleep disturbances. Although oral medications can mitigate motor symptoms, progressive motor fluctuations and levodopa-induced dyskinesias can exacerbate caregiver burden in PD. Advanced therapies such as enteral carbidopa/levodopa and DBS have the potential to improve refractory symptoms, but only enteral carbidopa/levodopa has been consistently associated with reductions in caregiver burden. Thus, the effective management of PD symptoms remains an unmet need in PD care that contributes to caregiver burden.

Caregiver-related factors also increase risk of burden. For example, female spouses and caregivers of male PD patients with dementia are more likely to experience heightened strain, suggesting that clinicians should be more cognizant of identifying caregiver burden in these populations. Although prior work has emphasized sex differences in caregiving, little is known about cultural or geographic differences in the caregiving experience that may influence caregiver outcomes. During the COVID-19 pandemic, caregivers also reported increased levels of anxiety and stress that were likely exacerbated by social isolation, home maintenance duties, and disruptions in access to healthcare and other support services.^124^ As pandemic restrictions ease, clinicians should ensure that caregivers have access to adequate patient and caregiver resources.

The emotional and psychological aspects of caregiving are particularly important. In qualitative studies, PD caregivers reported many losses, including those related to personhood and personal relationships with fewer opportunities for self-care. Additionally, social stigma and lack of coping skills increased caregiver strain and led to delays in seeking care. Interventions that address unmet caregiver needs, such as those providing psychosocial support, respite care, or targeted assistance for unequal gender dynamics,^125^ are critical to combat burden.

Fortunately, emerging evidence suggests that cognitive therapies and psychoeducational interventions can provide support and teach coping strategies to reduce caregiver burden in PD. Self-management programs are least effective at reducing burden and may impose additional tasks on caregivers. However, the 12 non-medical interventions featured in this review have important limitations, including small sample sizes, variable study designs, inconsistent outcome metrics, and lack of longitudinal data. Thus, more robust studies are needed to explore interventions that provide additional caregiver support without placing undue burden on caregivers, like outpatient palliative care initiatives. More recent data also suggests that caregivers may benefit from general and PD-specific support groups and helplines,^126^ though the availability of these resources can vary and have not been studied systematically. Current interventions are summarized in Table 1 to serve as a guide for clinicians and researchers. Future studies with more rigorous research methodology, such as RCTs, are critical to facilitate comparisons between caregiver interventions and/or standard care. These studies are currently lacking and represent a major knowledge gap in the field of PD.

In addition to the challenges noted above, we encountered several limitations when conducting this scoping review. First, we included English-language studies from 26 countries of origin: Australia, Brazil, Canada, China, Ethiopia, France, Germany, India, Iran, Israel, Italy, Japan, Malaysia, Mexico, Netherlands, New Zealand, Norway, Pakistan, Poland, Singapore, South Korea, Spain, Sweden, Turkey, the United Kingdom, and the US. Because cultural factors can contribute to perceptions of caregiver burden, it may be difficult to compare results drawn from different populations. Second, although all included studies explored caregiver burden in PD, some studies incorporated caregiver well-being into their discussion without differentiating between these outcomes. Caregiver burden and caregiver well-being are often considered distinct constructs.^127^ Although caregiver burden can directly impact caregiver well-being, the latter is more strongly associated with perceived social support, suggesting that research on caregiver burden should be supplemented with an emphasis on quality of life.^127^ Future research on this topic should take extra care to differentiate between caregiver burden and well-being if possible.

Because PD has no known cure, caregivers will continue to experience strain in caring for patients with advancing symptoms. To identify caregiver burden in PD, clinicians must routinely screen for burden and work with multi-professional teams to provide coping strategies and resources for those in need. After identifying burden, more effective and sustainable interventions are needed to manage burden and its associated complications. Caregiver burden remains an important and often overlooked challenge in PD care; improved recognition and treatment will not only lead to improved health outcomes for patients and their caregivers, but also reduce the societal and economic burden of caregiving.
